# Active smoking in urban households: An association between urinary cotinine metabolite level and serum eGFR concentration

**DOI:** 10.18332/tid/186071

**Published:** 2024-04-05

**Authors:** Jadsada Kunno, Titaporn Luangwilai, Panu Pimviriyakul, Saowanee Sematong, Busaba Supawattanabodee, Sathit Kuratong, Mark Gregory Robson

**Affiliations:** 1 Department of Research and Medical Innovation, Faculty of Medicine Vajira Hospital, Navamindradhiraj University, Bangkok, Thailand; 2 Department of Biochemistry, Faculty of Science, Kasetsart University, Bangkok, Thailand; 3 College of Public Health Sciences, Chulalongkorn University, Bangkok, Thailand; 4 Department of Medicine, Faculty of Medicine Vajira Hospital, Navamindradhiraj University, Bangkok, Thailand; 5 School of Environmental and Biological Sciences, Rutgers University, New Brunswick, United States

**Keywords:** active smoking, cotinine metabolite, kidney function, urban households

## Abstract

**INTRODUCTION:**

Smoking stands as a primary contributor to preventable deaths globally and is linked to an increased risk of developing kidney failure and other diseases. A few studies have focused on the negative correlation between serum cotinine and estimated glomerular filtration rate (eGFR), indicating decreased kidney function. This study investigated the associations between urinary cotinine metabolite concentration and serum eGFR among active smokers in urban households.

**METHODS:**

This was a cross-sectional study of active smokers in urban households’ community Bangkok, Thailand from January to April 2023. The study involved 85 participants aged ≥18 years who were active smokers. Both urinary cotinine and serum eGFR concentrations were used as biomarkers. Independent sample t-tests were used to compare the urinary cotinine metabolite based on differences in the characteristic variable. We used multiple linear regression to test the association between cotinine metabolite and characteristics variables. Spearman’s analysis was used to test the correlation between cotinine metabolite and eGFR concentration.

**RESULTS:**

The association between urinary cotinine metabolite and serum eGFR concentration decreased with increasing cotinine concentrations (r= -0.223, p=0.041), suggesting a decline in kidney function. However, this study found no significant difference between urinary cotinine metabolite and characteristic variables (p>0.05). Additionally, those who smoked for ≥10 years (117.40 ± 89.80 ng/mL), smoked ≥10 cigarettes per day (117.40 ± 89.80 ng/mL) and used conventional cigarettes (124.53 ± 115.10 ng/mL). The results of the multiple linear regression models analysis indicated that those who were smokers for ≥10 years (β=0.076; 95% CI: -31.575–59.715) and those who were smoked ≥10 cigarettes/day (β=0.126; 95% CI: -65.636–18.150) were not associated with urinary cotinine metabolite level.

**CONCLUSIONS:**

This study shows that the urinary cotinine metabolite level is associated with serum eGFR concentration among active smokers in urban households. The current study suggests that clinical identification and a prospective cohort study are needed before robust conclusions about how tobacco affects kidney efficiency.

## INTRODUCTION

Tobacco use or smoking increases the risks of infection or the severity of some communicable diseases in global public health^1^. Smoking, a well-known risk factor for many diseases, is a risk factor for the development and progression of chronic kidney diseases (CKDs) in communities^2^.

The mechanisms of smoking-related renal damage, including its vascular and tubular effects, are unknown to most smokers^3^. Smoking-induced oxidative stress leads to endothelial dysfunction, decreased estimated glomerular filtration rate (eGFR), and biochemical evidence of smoking or chronic cotinine-induced renal toxicity, even in the absence of histological changes^4,5^. The causes of CKDs are heterogeneous. Studies showed that the relationship between cigarette smoking and kidney impairment depends on the underlying kidney disease^6^. Numerous studies also indicate a relationship between smoking and kidney function^7-11^. The relative risk of developing gross proteinuria (>300 mg/day) during four years of observation was 2–2.5-fold higher in heavy smokers when compared to never smokers^12^.

Nicotine is the main component of tobacco smoking, but its short half-life (1–3 hours) has limited value as a marker of exposure. Nicotine is mainly transformed to cotinine (active metabolite), the primary proximate metabolite of nicotine, which is used most frequently as a biomarker of tobacco smoke exposure, as its half-life is longer (approximately 16–18 h), and its levels remain relatively constant during the day^13-16^. Cotinine is a good quantitative test for the detection of smoking exposure that can be measured in different body fluids, including saliva, plasma, and urine^17^.

In Thailand, the current reduction in tobacco smoking is insufficient to achieve a 30% reduction in the prevalence of tobacco smoking by 2025^18^. Tobacco consumption is one of the major risk factors for non-communicable diseases (NCDs) as a whole; one in six deaths from NCDs is related to tobacco^19^. In addition, smoking leads to a decrease in kidney function in adults^20^. Moreover, some studies demonstrated a strong statistically significant association between secondhand and impaired renal function^21^. Furthermore, a few studies showed a negative correlation between urinary cotinine metabolite and serum eGFR, indicating decreased kidney function^22^. Based on the previous evidence, our study sought to understand whether cigarette smoking is associated with kidney function. Our hypothesis posits that increased cotinine metabolite levels will affect kidney function, based on eGFR, and that these can act as biomarkers for active smoking in urban households in Thailand, where tobacco consumption needs to steadily decrease^23,24^. Few studies have investigated the association between cotinine metabolite levels and serum eGFR concentrations among active smokers in urban households in Thailand. Specifically, our study aimed to: 1) analyze urinary cotinine metabolite and serum eGFR levels among active smokers; 2) compare urinary cotinine metabolite and characteristics variables; 3) establish associations between urinary cotinine metabolite and characteristics variables; and 4) establish a correlation between urinary cotinine metabolite and serum eGFR concentration among active smokers.

## METHODS

### Study design

This was a cross-sectional survey of active smokers from January to April 2023. The study was approved by the Faculty of Medicine Vajira Hospital ethics committee, Navamindradhiraj University, Bangkok, Thailand (COA 067/2564). All experiments were performed in accordance with relevant guidelines and regulations specified in the Declaration of Helsinki.

### Participants

This study was conducted using purposive sampling of smokers who live in a slum-urban household community in Bangkok because it has a high prevalence of active cigarette smokers. All participants were chosen by convenience sampling. Participants were asked to confirm their voluntary participation and provided instructions for completing the questionnaire. The included smokers were aged ≥18 years, and participants underwent a complete clinical examination. A comprehensive history was taken, with particular emphasis on the age and sex of those who currently smoked at least five cigarettes per day (average 150 cigarettes per month or 1800 cigarettes per year). Our study sample was representative of active cigarette smokers in household areas, including indoor and outdoor households in the slum household community in Bangkok.

The definition of an active cigarette smoker in this study was calculated using the smoking index (SI) as the number of packs smoked daily multiplied by the number of years of smoking. The SI was used to categorize active smokers as: 1) mild active cigarette smokers (SI<400 cigarettes/year), 2) moderate active cigarette smokers (SI = 400–800 cigarettes/year), and 3) heavy active cigarette smokers (SI ≥800 cigarettes/year)^17,23^. The National Center for Health Statistics 2017 defined a current smoker as a person who has smoked ≥100 cigarettes in their lifetime and who is still smoking cigarettes^17^.

The sample size was calculated using G*Power (the effect size is 0.35) based on the estimated population of active cigarette smokers in the selected community. The Institutional Review Board of the Faculty of Medicine Vajira Hospital is in full compliance with the international guidelines for human research protection as the Declaration of Helsinki, The Belmont Report, the CIOMS Guideline, and the International Conference on Harmonization in Good Clinical Practice (ICH-GCP). All participants in this study provided written informed consent before joining the study. The study was performed in accordance with the Declaration of Helsinki and was approved by an appropriate ethics committee.

### Questionnaire

The questionnaire, divided into two sections, took about 10 minutes to complete (Supplementary file). The first part contained questions about the background of the participants, including age, status, occupation, education, weight (kg), height (cm), and disease status. The second section queries the behaviors of the smokers, including the duration of smoking, frequency of cigarettes per day, frequency of smoking per week, period of smoking, type of cigarettes used, the place where they smoked, their reason for smoking, the opportunity to quit smoking, how they would like to be helped to quit smoking, and interest in using a mobile application for quitting smoking. The questions were designed and modified by an expert team of researchers, and the content validity score was 0.85

### Data collection

All measurements were done after the participants provided written consent and had answered the questionnaire. Each participant provided a 50 mL urine sample during our visits to the community center. The urine samples were collected in standard polypropylene specimen containers. Then, they were put into the tube in a zip-lock plastic bag, kept in an ice box during transportation to the laboratory, and stored at -20°C until they were analyzed, which occurred within 24 hours.

Additionally, each participant provided 3 mL of blood samples to analyze kidney function. The blood samples were collected in a serum separator tube. The sample was stored under an ice bag following a blood drawing until it was transferred to the laboratory. Kidney function analysis was stored at 2–8°C until the analysis was done.

### Urinary cotinine metabolite analysis

The urine samples were thawed to analyze the urinary cotinine metabolite using an enzyme-linked immunosorbent assay kit (ELISA). Briefly, the cotinine ELISA process of the urine sample of each smoker (n=85) followed the instructions of the kit (catalog number KA0930, 96 assays, v.08, Abnova USA). The resulting curves appeared linear between 0 and 100 ng/mL of the cotinine concentration. The determination of recovery for the cotinine concentration analytes was 99% in a urine sample at the standard concentration provided in the cotinine ELISA kit. Additionally, we followed and modified all the methods from previous studies^24-28^.

### Serum eGFR analysis

The blood samples were thawed to analyze the kidney condition of the participants based on serum eGFR. The analysis was done using the laboratory provided by the Faculty of Medicine Vajira Hospital, Navamindradhiraj University, Bangkok, Thailand.

The outcome variable, kidney disease stage, was defined based on the eGFR (mg/minute/1.73 m^2^) as follows: normal range, ≥90; early kidney disease, 60–89; kidney disease, 15–59; and kidney failure, <15^22,29-32^.

### Statistical analysis

In addition to descriptive statistics of the characteristics and dispersion measures (mean and standard deviation), independent sample t-test and one-way analysis of variance (F-test) were used to compare the urinary cotinine metabolite based on differences in the characteristic variable. Multiple linear regression was used to test the association between urinary cotinine metabolite and the characteristics variables. Spearman’s correlation analysis examined the correlation between urinary cotinine metabolite and serum eGFR concentration. The level of statistical significance was set at p<0.05. The statistical analysis was performed using the Statistical Package for the Social Sciences Program (SPSS), version 28 (IBM Corp., Armonk, NY, USA).

## RESULTS

### Active smoking characteristics

Of the included participants, 51.8% were males and 48.2% were females. Overall, 50.6% of the participants were younger than 37 years, and most were married (87.1%), employees (83.5%), and had an education level lower than elementary school (52.9%). The average age of the participants was 37 years; their average weight was 61.7 kg, 76.5% of whom had healthy weight [body mass index (BMI) ranging 18.5–24.9 kg/m^2^]; and 83.5% had no prior disease (Table 1).

**Table 1:** Characteristics of active smoking behavior and biomarkers identification (N=85)

*Characteristics*	*n*	*%*
**Smokers’ information**		
**Gender**		
Male	44	51.8
Female	41	48.2
**Age** (years)		
Mean ± SD	37.00 ± 13.50	
<37	43	50.6
≥37	42	49.4
**Marital status**		
Single	11	12.9
Married	74	87.1
**Occupation**		
Unemployed	14	16.5
Employed	71	83.5
**Education level**		
Lower than elementary school	45	52.9
Higher than elementary school	40	47.1
**Weight** (kg)		
Mean ± SD	61.70 ± 8.90	
**Height** (cm)		
Mean ± SD	162.24 ± 7.73	
**BMI** (kg/m^2^)		
Mean ± SD	23.43 ± 2.97	
<18.5 (underweight)	3	3.5
18.5–24.9 (normal weight)	65	76.5
25–29.9 (overweight)	14	16.5
≥30 (obese)	3	3.5
**Disease**		
None	71	83.5
Disease	14	16.5
**Active smoking behavior**		
**Duration of smoking** (long-term exposure) (years)		
<10	39	45.9
≥10	46	54.1
**Cigarettes/day**		
<10^a^	35	41.2
≥10^b^	50	58.8
**Place for smoking in the household**		
Indoors	72	82.8
Outdoors	13	14.9
**Type of cigarettes**		
Instant cigarette	8	9.4
Self-rolled cigarette	77	90.6
**Reason for smoking**		
Relaxation and happiness	65	76.5
Other	20	23.5
**Opportunity to quit smoking**		
Not sure	61	71.8
Trend to quit smoking	24	28.2
**Would you like to be helped to quit smoking?**		
Quit smoking by yourself	81	95.3
Other	4	4.7
**Are you interested in using a mobile application to quit smoking?**		
Not interested	9	10.6
Interested	76	89.4
**Biomarkers**		
**Kidney function**		
**Serum eGFR** (mL/min/1.73 m^2^)		
Mean ± SD	85.48 ± 1.64	
Range	33.60–105.80	
<90	66	77.6
≥90	19	22.4
**Urinary cotinine metabolite** (ng/mL)		
Mean ± SD	103.85 ± 10.09	
Range	10.28–297.29	

a Cigarette consumption: 5–10 cigarettes/day (average, 1800–3600 cigarettes/year).

b Cigarette consumption: ≥10 cigarettes/day (average, ≥3600 cigarettes/year).

In terms of smoking behavior, most of the participants had smoked for >10 years (54.1%), and over 58.8% smoked more than 10 cigarettes daily (average ≥15 packs/month or average ≥180 packs/year, and average 3600 cigarettes/year). Most of the participants used self-rolled cigarettes (68.2%). Most of the participants were smoking indoors in households (82.8%) (Table 1).

Most participants smoked because it helped them to relax and made them happy (76.5%). Moreover, the majority of participants were uncertain about quitting smoking (71.8%). However, they would quit smoking by themselves (95.3%) and were interested in using a mobile application to quit smoking (89.4%) (Table 1).

### Urinary cotinine metabolite and kidney function as biomarkers for smoking exposure

Regarding urinary cotinine metabolite as a biomarker, the mean ± SD urinary cotinine metabolite among extreme smokers was 103.85 ± 10.09 ng/mL (range: 10.28–297.29) (Table 1).

The participants’ median ± SD serum eGFR was 81.70 ± 12.35 mL/min/1.73 m^2^ (range: 33.60–105.80) (Table 1). Among the active smokers, 77.6% had serum eGFR <90 mL/min/1.73 m^2^, with their eGFRs ranging from 60–89 mg/minute/1.73 m^2^.

### Comparison between urinary cotinine metabolite and characteristics variables

Table 2 presents the comparison of urinary cotinine metabolite among active smokers. However, this study found no significant difference between urinary cotinine metabolite and characteristics variables (p>0.05). Regarding gender, both male and female show similarity (103.97 ± 97.97 ng/mL) and (103.73 ± 88.74 ng/mL), respectively. Among active smokers, the majority were aged ≥37 years (109.85 ± 88.37 ng/mL), had an education level lower than elementary school (116.25 ± 93.15 ng/mL), smoked for ≥10 years (117.40 ± 89.80 ng/mL), smoked ≥10 cigarettes per day (117.40 ± 89.80 ng/mL) and used conventional cigarettes (124.53 ± 115.10 ng/mL).

**Table 2:** Comparison of urinary cotinine metabolite among active smoking behavior

*Characteristics*	*Comparison of urinary cotinine metabolite (ng/mL)*		
	*Mean ± SD*	*t/F*	*p*
**Smokers’ information**			
**Gender**			
Male	103.97 ± 97.97	0.012	0.156
Female	103.73 ± 88.74		
**Age** (years)		-0.584	0.336
<37	98.00 ± 98.14		
≥37	109.85 ± 88.37		
**Marital status**			
Single	115.91 ± 79.15	0.458	0.169
Married	102.06 ± 95.32		
**Occupation**			
Unemployed	106.33 ± 91.49	0.108	0.675
Employed	10.337 ± 94.02		
**Education level**			
Lower than elementary school	116.25 ± 93.15	1.308	0.874
Higher than elementary school	89.91 ± 92.16		
**BMI** (kg/m^2^)			
<18.5 (underweight)	106.60 ± 83.50	0.126	0.945
18.5–24.9 (normal weight)	100.51 ± 96.47		
25–29.9 (overweight)	116.93 ± 72.21		
≥30 (obese)	112.57 ± 153.37		
**Disease**			
None	97.61 ± 93.28	-1.400	0.384
Disease	135.51 ± 88.53		
**Active smoking behavior**			
**Duration of smoking** (long-term exposure) (years)			
<10	96.79 ± 93.56	-0.642	0.913
≥10	109.85 ± 93.28		
**Frequency of cigarettes/day**			
<10^a^	94.37 ± 95.04	1.125	0.342
≥10^b^	117.40 ± 89.80		
**Place for smoking in the household**			
Indoors	104.91 ± 90.73	0.244	0.195
Outdoors	98.03 ± 109.05		
**Type of cigarettes**			
Instant cigarette	124.53 ± 115.10	0.658	0.315
Self-rolled cigarette	101.71 ± 91.13		

a Independent t-test.

b One-way analysis of variance (ANOVA).

* Statistically significant level p<0.05. Urinary cotinine metabolite (ng/mL): mean ± SD: 103.85 ± 10.09, range: 10.28–297.29.

### Associations between urinary cotinine metabolite and characteristics variables

The multiple linear regression models analysis results indicated no significant association between urinary cotinine metabolite and the characteristics variables (Table 3). Female (vs Male, β= -0.003; 95% CI: -47.806–46.54) and aged ≥37 years (vs Male, β= -0.003; 95% CI: -34.972–52.190) were not associated with urinary cotinine metabolite level.

**Table 3:** Multivariate analysis of urinary cotinine metabolite and factor variables

*Variables*	*β (95% CI)*	*p*
**Smokers’ information**		
**Gender**		
Male ^®^		0.979
Female	-0.003 (-47.806–46.54)	
**Age** (years)		
<37 ^®^		0.695
≥37	0.047 (-34.972–52.190)	
**Extreme smoker**		
**Duration of smoking** (long-term exposure) (years)		
<10 ^®^		0.541
≥10	0.076 (-31.575–59.715)	
**Cigarettes/day** (extremely heavy active cigarette smoker)		
<10 ^®^		0.263
≥10	0.126 (-65.636–18.150)	

Data were analyzed using the multiple linear regression models.

* Statistically significant level p<0.05.

^®^ Reference categories.

Those who were smokers for ≥10 years (vs those who were smokers for <10 years (β=0.076; 95% CI: -31.575–59.715) and those who smoked ≥10 cigarettes/day (vs those who smoked <10 cigarettes/day, β=0.126; 95% CI: -65.636–18.150) were not associated with urinary cotinine metabolite level.

Correlation between urinary cotinine metabolite and serum eGFR concentration

Table 4 indicates a weak but significant negative correlation between urinary cotinine metabolite and serum eGFR concentration (r= -0.223, p=0.041). However, eGFR decreased with increasing cotinine concentration, suggesting a decline in kidney function.

**Table 4:** Spearman correlation analysis between urinary cotinine metabolite and serum eGFR

Biomarkers	Correlation coefficient (r)	
	Urinary cotinine metabolite (ng/mL)	p
Urinary cotinine metabolite (ng/mL)	1	-
Serum eGFR (mL/min/1.73 m^2^)	-0.223	**0.041**

Correlation coefficient significant at p<0.05.

## DISCUSSION

This study was conducted while developing health policies for tobacco prevention and control in Thailand. Our study was performed among active smokers in a slum-urban household community in Bangkok. Among the participants, 67.1% smoked ≥10 cigarettes/day (SI ≥3600 cigarettes/year), 32.9% smoked 5–10 cigarettes/day (average: 1800–3600 cigarettes/year) and 90.6% used self-rolled cigarettes.

The preliminary results showed that the majority of the participants were uncertain about quitting smoking (71.8%). However, they would quit smoking by themselves (95.3%) and were interested in using a mobile application for quitting smoking (89.4%). Thus, this result will promote future research to develop a mobile application for quitting smoking in an urban community.

### Urinary cotinine metabolite and serum eGFR levels among active smokers

Our study found a urinary cotinine level of ≥100 ng/mL amongst active smokers, as in a prior study^33^. In addition, cotinine levels among Northern Americans and Southwest Americans were 81.6 ng/mL and 44.8 ng/mL, respectively^34^, while other studies found 89.5 ng/mL in smokers^35^. Hence, our study answers the questions about increased cotinine metabolite levels among active smokers in urban households. Thus, our results could further support prevention strategies from local policymakers.

In terms of serum eGFR levels, the mean ± SD eGFR of the participants was 85.48 ± 1.64 mL/min/1.73 m^2^, suggesting kidney damage with mild loss of kidney function based on the criteria to define CKDs^31^: the average eGFR ranges 60–89 mL/min/1.73 m^2^. Additionally, the average age of our participants was 37 years. The normal range of eGFR number is usually >90 mL/min/1.73 m^2^; the average among individuals aged 30–39 years^31^ is 107 mL/min/1.73 m^2^. Thus, based on our findings, smoking, and age >37 years might affect serum eGFR concentrations. Cigarette smoking is a key modifiable risk factor for renal disease^10,29,30^. Hence, smoking can affect kidney functions and cause early-stage kidney disease. Many factors, including age and gender may influence kidney function as an indicator of early kidney disease. As expected, an age-dependent decrease of eGFR is observed in both genders. In cross-sectional datasets, the median eGFR decrease is approximately 110 mL/min/1.73 m^2^ among individuals aged 35 years^34^. Our study suggests that longitudinal studies are needed before definitive conclusions can be reached that smoking can affect kidney functions.

### Comparison between urinary cotinine metabolite and characteristics variables

According to various characteristics, this study hypothesized different urinary cotinine metabolite concentration among active smokers. However, this study found no significant difference between urinary cotinine metabolite and characteristic variables. Although most other research used questionnaires for determining smoking exposure^36^, our study used direct smoking exposure detection. In addition, our study found that both genders had the same urinary cotinine metabolite concentration, in contrast to a previous study that found higher concentrations in males than in females^37^. Our findings showed that those with lower education level than elementary school and aged >37 years showed higher urinary cotinine metabolite level, which might be important risk factors for smoking^38^. Moreover, this study found that those who were unemployed, used conventional cigarettes, and smoked ≥10 cigarettes per day showed higher urinary cotinine metabolite levels.

### Associations between urinary cotinine metabolite and characteristics variables

The multiple linear regression model analysis showed gender, age, duration of smoking, and frequency of cigarettes per day were not associated with the urinary cotinine metabolite level. Although it was not described in this study, we found that inadequate support for health warrants surveillance to protect people from major risk factors and prevention strategies from local policymakers. Moreover, a previous study found heavy smoking (>30 packs/year) to be an important risk factor for the development of chronic kidney disease^39^. Recommendations should focus on the importance of smoking cessation to decrease the incidence of kidney disease and other preventable diseases such as coronary artery diseases and cancers. Hence, strategies should focus on reducing fear, improving attitudes toward the care of smokers, and promoting preventive practices. Therefore, we suggest education, increased awareness, and management to reduce the risk of long-term smoke exposure in households. Our study is supported by a previous study in Thailand, where the smoking prevalence in males decreased from 40.8% in 2015 to 37.8% in 2025^18^. However, the findings of a previous study were that females metabolize cotinine faster than males, affecting their cigarette uptake. Smoking among men and women affects cotinine levels and measures of physical or psychological dependence, but these are underexplored in most studies^37,40-42^.

### Correlation between urinary cotinine metabolite and serum eGFR concentration

Our study aligns with previous research in which eGFR decreased with increasing cotinine concentrations, which suggests a decline in renal function^22^. On the contrary, the effects of cigarette smoking on the kidneys in healthy individuals without established renal diseases have not been established^43^. Moreover, other studies recommended that chronic kidney disease is correlated with smoking exposure and suggested its potential utility in clinical trials examining changes in smoking behavior and effects on kidney disease^26^. On the other hand, it was found that urine cotinine might be associated with a 1.6-fold higher risk of incident atrial fibrillation in nonsmokers^27^.

Additionally, a previous study showed that among smokers, higher serum concentrations of cotinine were significantly associated with serum creatinine compared with non-smokers^17^. Our results are similar to those of a previous study, which showed higher levels of creatinine-based eGFR and lower levels of cystatin C-based eGFR with higher quantities of smoking^11^. Smoking might affect renal function by causing chronic endothelial dysfunction, oxidative stress, and hardening of the glomeruli^44-46^. Moreover, exposure to passive smoking has been reported to be associated with significantly increased lipid peroxidation in the liver, increased catalase activity in the kidney, cardiac and blood vessel diseases, abnormal renal blood flow, and renal afflictions over time^38^. However, a few studies have identified a non-linear relationship between smoking and renal function in different populations through stratified analysis^22^. Hence, urinary cotinine metabolite measurement can be used as a key to providing targeted feedback in interventions for smoking/quitting populations to promote smoking behavior changes. Our future goal is to develop health policies for the surveillance of tobacco prevention and control in urban communities in Thailand.

### Limitations

This cross-sectional study involved data collection at a single time point, which may introduce selection bias. The number of subjects was too small when selected by active smokers. A larger number of smokers or non-smokers should be included in future studies. Recommendations for future studies include matching smokers and non-smokers based on demographics and other factors. Additional research should be conducted on the purported benefits of quitting smoking on kidney function.

## CONCLUSIONS

This study examined the potential correlation of increased cotinine metabolite levels among smokers and kidney function, as indicated by eGFR. We found that increased urinary cotinine metabolite levels were correlated with decreased eGFR concentration among active smokers. These can act as biomarkers for active smoking for those living in slum households and contribute in developing health policies for tobacco prevention and control in urban communities in Thailand. We recommend researching the benefits of quitting smoking on kidney function and how it can guide public health strategies.

## Supplementary Material



## Data Availability

The data supporting this research are available from the authors upon reasonable request.
