# Supplementary material for: Active smoking in urban households: An association between urinary cotinine metabolite level and serum eGFR concentration
Source: Tob Induc Dis. 2024 Apr 5;22:10.18332/tid/186071. doi: 10.18332/tid/186071 (PMC10996036; doi:10.18332/tid/186071)
Supplement: Supplementary file 1 [file TID-22-59-s1.pdf]

Supplementary file 1

Title: Active smoking in urban households: An association between urinary cotinine metabolite and serum eGFR concentration

The full English language version of the questionnaire. The full English language version of the questionnaire contained all the details of the original

Questionnaire (English version)

All participating in this study provided written informed consent from before

( ) Allow ( ) Not allow

ID ....

The questionnaire is divided into two sections.

Section 1. Smokers' information

Age

....

Status

( ) Sigle

( ) Others

Occupation

( ) Non-work

( ) Other

Education

( ) lower than elementary school

( ) higher than elementary school

Weight (kg)

....

High (cm)

....

Disease

( ) Non-disease

( ) Disease

Section 2. Parent information

Duration of smoking

( ) < 10 years

( ) ≥ 10 years

Volume of cigarettes/day

( ) ≤ 10 cigarettes

( ) ≥ 10 cigarettes

Frequency of smoking/week

( ) 4 – 6 days

( ) Everyday

Time period of smoking

( ) Some time

( ) Every time

Place for smoking

( ) Indoor household

( ) Outdoor household

Type of cigarettes

( ) Instant cigarette

( ) Self-rolled cigarettes

Reason for smoking

( ) Relax and happiness

( ) Others

Opportunity to quit smoking

( ) Not sure

( ) Trend for quit smoking

How would you like to help you quit smoking?

( ) Quit smoking by yourself

( ) Other

Are you interested in using a mobile application for quit smoking?

( ) Not interested

( ) Interested

Urine collection 50 mL per person

( ) Allow ( ) Not allow

blood collection 3 mL per person

( ) Allow ( ) Not allow

© 2024 Kunno J. et al.
